# Quality of life, stress, anxiety and depression and associated factors among people with type 2 diabetes mellitus in Western region Saudi Arabia

**DOI:** 10.3389/fpsyt.2023.1282249

**Published:** 2024-01-15

**Authors:** Shahad Abduljalil Abualhamael, Mukhtiar Baig, Waleed Alghamdi, Zohair Jamil Gazzaz, Majid Al-Hayani, Abdulrahman Bazi

**Affiliations:** ^1^Department of Internal Medicine, Faculty of Medicine, Rabigh, King Abdulaziz University, Jeddah, Saudi Arabia; ^2^Department of Clinical Biochemistry, Faculty of Medicine, Rabigh, King Abdulaziz University, Jeddah, Saudi Arabia; ^3^Department of Psychiatry, Faculty of Medicine, King Abdulaziz University, Jeddah, Saudi Arabia; ^4^Department of Internal Medicine, Faculty of Medicine, Rabigh, King Abdulaziz University, Jeddah, Saudi Arabia

**Keywords:** diabetes mellitus, quality of life, stress, anxiety and depression, RV-DQoL13, DASS-21 diabetes mellitus, mental health, DASS-21

## Abstract

**Introduction:**

The objective of this study is to evaluate the quality of life (QoL), depression, anxiety, and stress, along with associated factors among individuals with diabetes in Saudi Arabia.

**Methods:**

This survey was conducted at King Abdulaziz University (KAU), Jeddah, Kingdom of Saudi Arabia (KSA). The assessment of depression, anxiety, and stress related to Type 2 Diabetes Mellitus (T2DM) was conducted using the DASS-21 questionnaire, while diabetes-related QoL was evaluated using the revised version of the diabetes QoL questionnaire (RV-DQoL13). Data were analyzed using SPSS-26.

**Results:**

A total of 251 subjects were included in the study (165 [65.7%] males and 86 [34.3%] females, mean age 50.1 ± 14.5 years). The individuals with DM had a mean value of QoL of 29.16 ± 9.23, with 46.9% having poor QoL. Furthermore, in dimensions of QoL, almost half of the individuals reported high worry about the disease (49.6%), followed by a high diabetes impact (46.6%) and low life satisfaction (42.9%). The prevalence of depression, anxiety, and stress was 49.4, 71.7, and 49.8%, respectively. A significant correlation was found between depression, anxiety, and stress and DASS-21 scores with QoL (*p* < 0.001). The regression analysis indicated an association of distinct factors with QoL like age above 41 years (*p* = 0.004), being married (*p* < 0.001), being divorced (*p* = 0.04), higher education (*p* = 0.007), regular medicine intake (*p* = 0.01), regular exercise (*p* = 0.03), lipid profile (*p* = 0.01), HbA1c (*p* < 0.001), and DASS-21 scores (*p* < 0.001). Poor QoL score (TQoL score > 27) was significantly associated with depression, anxiety, and stress (*p* < 0.001). The participants with higher monthly income, shorter disease duration, regular medicine use, and altered lipid profile, and older subjects had a lower chance of depression, anxiety, and stress.

**Conclusion:**

Approximately half of individuals with T2DM experienced poor QoL, while the prevalence rates for depression, anxiety, and stress were 49.4, 71.7, and 49.8%, respectively. Scores in the domains of impact, worry, and satisfaction were below optimal levels. Several factors were linked to QoL and depression, anxiety, and stress, and an association was observed between DASS-21 scores and QoL.

## Introduction

Quality of life (QoL) constitutes a pivotal measure for assessing the health consequences of managing chronic conditions like type 2 diabetes mellitus (T2DM), reflecting both the physical and psychosocial burdens faced by patients (1). QoL provides insight into the patient’s perspective on illness outcomes and contributes valuable information to medical and epidemiological data (2). The primary goal of diabetes management is to enhance patients’ health-related quality of life (HRQoL) (3). T2DM comprises a complex spectrum of metabolic disorders that present significant challenges to healthcare systems and communities (4). Projections suggest that by 2035, approximately 592 million individuals worldwide could be affected by T2DM (5).

Research demonstrates that T2DM is a multifaceted disorder with associations to age, gender, ethnicity, diabetes control status (glycemic control), educational level, depression and anxiety, sedentary lifestyles, and a positive family history of T2DM (6). T2DM is linked to reduced QoL, heightened T2DM-related healthcare costs, and decreased life expectancy (7). In Saudi Arabia, increased physical inactivity, consumption of unhealthy diets, obesity, and sedentary lifestyles contribute to T2DM’s escalating healthcare burden (8, 9). Saudi Arabia ranks highest in age-adjusted DM prevalence (17.7%) in the MENA region and fourth globally in diabetes prevalence (10).

Epidemiological studies indicate that approximately one-third of individuals with T2DM experience depression or anxiety (5). Extensive literature establishes a robust correlation between diabetes, depression, and anxiety. The frequency of anxiety and depression among T2DM patients significantly exceeds that of the non-T2DM population, ranging from approximately 12 to 28% (11). In developing countries, estimates suggest that up to 45–58% of T2DM outpatients have anxiety or depression. These psychological conditions are independently associated with higher mortality and morbidity in T2DM patients (12). Conversely, depression and anxiety also negatively impact T2DM status, exacerbating the condition’s negative outcomes (12, 13).

Existing data indicates that the presence of diabetes and depression diminishes QoL and contributes to financial, functional, and psychological stresses (11). Research highlights that depression among individuals with diabetes hampers adherence to exercise, dietary plans, social interactions, and medication regimes. Emotional disturbances, including anxiety and depression, pose a significant threat to QoL in most individuals with T2DM. Emotional stress is linked to suboptimal glycemic control and self-care practices (12).

Given diabetes’ pronounced impact on QoL and the multifaceted nature of QoL, which encompasses physical, social, and emotional dimensions related to the illness and its treatment (14), it becomes crucial to identify factors that detrimentally affect the QoL of individuals with diabetes and to design interventions that target improvements in their QoL (15). The literature reveals divergent results concerning these factors. Moreover, although numerous studies have separately evaluated depression, anxiety, and stress and QoL among individuals with T2DM, the simultaneous assessment of both remains scarce. Therefore, our study investigated the QoL, depression, anxiety, and stress, and their associated factors among Saudi inhabitants with T2DM using the DQoL and DASS-21 instruments.

## Methods

This cross-sectional study utilized two questionnaires and was conducted at the Faculty of Medicine, Rabigh, KAU, Jeddah, KSA, between September 2021 and March 2022. Ethical approval for the study was obtained from the Biomedical Ethics Research Committee of KAU, Jeddah (Reference No. 449–19). The study adhered to the principles of the Helsinki Declaration, and informed consent was obtained from each participant before data collection.

A non-probability convenient sampling strategy was employed. The study included individuals diagnosed with T2DM both genders and aged between 25 and 65 years. Exclusion criteria comprised individuals with Type 1 diabetes mellitus, cognitive impairment, physical disabilities, severe illness, pregnant women, and those who declined consent.

Baseline demographic information, such as age, gender, weight (in kg), height (in m2), BMI (kg/m^2^), educational background, and socioeconomic status, was recorded. Detailed data collection involved face-to-face interviews with individuals with diabetes, during which they completed two questionnaires—one gathering personal and demographic information and the other focusing on subject-specific information. The diabetes-associated depression, anxiety, and stress were measured using DASS-21 questionnaire, while the Revised Version of Diabetes Quality of Life (RV-DQoL13) questionnaire was used to evaluate diabetes-related QoL (16, 17).

The reliability and validity of the RV-DQoL13 questionnaire have been established (17, 18). However, since its Arabic version was unavailable, a bilingual expert translated the RV-DQoL questionnaire from English to Arabic to ensure accurate translation. The questionnaire’s construct validity was assessed by two medical educators. A pilot study involving 50 individuals with T2DM was conducted to assess questionnaire readability and comprehension. The questionnaire was further refined to enhance comprehension following discussions with the participants. Cronbach’s alpha was calculated for each section of the questionnaire, resulting in values of 0.84, 0.76, and 0.75 for satisfaction, impact, and worry, respectively. A validated Arabic version of the DASS-21 questionnaire was utilized (19).

The RV-DQoL13 questionnaire comprises 13 items, primarily focusing on three domains: satisfaction (6 items), impact (4 items), and worry (3 items). Participants responded to statements within each domain on a Likert scale ranging from 1 to 5. The maximum scores for the satisfaction, impact, and worry domains are 30, 20, and 15, respectively. The total score ranges from 13 to 65, where higher scores indicate poorer QoL. In the DQoL questionnaire, overall QoL was categorized as ‘low quality of life’ (DQoL score > population mean) or ‘good quality of life’ (total DQoL score < population mean). For domains, low life satisfaction, high diabetes impact, and high diabetes worry were determined based on satisfaction, impact, and worry scores exceeding the population mean, respectively. Median values were considered for all categories instead of mean values (20).

The DASS-21 questionnaire comprises 21 symptoms categorized into three subscales: anxiety, depression, and stress. These subscales measure three dimensions and exhibit relatively good reliability estimates (0.84 to 0.97) (16). Scores on the anxiety, depression, and stress subscales are categorized as normal, mild to moderate, and severe based on score ranges (0–7, 8–14, >14; 0–9, 10–20, >20; 0–14, 15–25, >25) respectively. While the scores do not provide a functional diagnosis, they indicate the potential presence of depression, anxiety, and stress.

### Statistical analysis

Data analysis was performed using SPSS version 26. In the RV-DQoL13 questionnaire, higher scores indicated poorer QoL, while higher scores in the DASS-21 questionnaire indicated increased depression, anxiety, and stress levels. Quantitative data were presented as percentages and frequencies, while qualitative data were expressed as mean and standard deviation. Pearson’s correlation test assessed the correlation between the DASS-21 scale and DQoL scores. Univariate and multivariate logistic regression analyses were employed to determine associations between various factors and DQoL and depression, anxiety, and stress. Binary categories were created based on median scores for QoL (TQoL score ≤ 27 for good QoL, TQoL score > 27 for low quality of life) and for depression, anxiety, and stress (depression, anxiety, and stress score ≤ 38 and > 38). Significance was determined if the value of p was less than 0.05.

## Results

A total of 251 subjects participated in the study, including 165 (65.7%) males and 86 (34.3%) females, with a mean age of 50.1 ± 14.5 years. Additional baseline characteristics are presented in Table 1.

**Table 1:** General characteristics of the study participants (*n* = 251).

Variables	Categories	Frequency (%)
Age (in years)	Mean ± SD	
	50.1 ± 14.5	
Gender	Male	165(65.7)
	Female	86(34.3)
Marital Status	Single	37(14.7)
	Married	194(77.3)
	Widowed	8(3.2)
	Divorced	12(4.8)
Education Level	No formal education	9(3.6)
	Primary	11(4.4)
	Middle	11(4.4)
	Secondary	45(17.9)
	Bachelor	96(38.2)
	Master	20(8.0)
	Ph. D.	16(6.4)
	Diploma	43(17.1)
Monthly Income	Less than five thousand SR	60(23.9)
	Between 5 thousand to 10 thousand SR	58(23.1)
	Between 10 thousand to 15 thousand SR	56(22.3)
	more than 15 thousand SR	77(30.7)
When were you Diagnosed?	Less than one year	31(12.4)
	One to 10 years	119(47.4)
	Ten to 20 years	74(29.5)
	More than 20 years	27(10.8)
Are you taking your medication regularly	Yes	215(85.7)
	No	36(14.3)
Are you Smoker?	Yes	62(24.7)
	No	189(75.3)
Do you exercise regularly	Yes	58(23.1)
	No	193(76.9)
Lipid profile in last report	Altered	94(37.5)
	Not altered	157(62.5)
Are you getting checked your lipid profile regularly	Yes	136(54.2)
	No	115(45.8)
HbA1c in last report	Elevated	124(49.4)
	Not elevated	127(50.6)

The mean scores for the satisfaction, impact, and worry domains were 13.14 ± 4.84, 9.2 ± 4.84, and 6.82 ± 2.78, respectively. Individuals with T2DM had an average DQoL score of 29.16 ± 9.23, with 46.9% experiencing poor QoL. Moreover, concerning DQoL, nearly half of the participants reported high levels of worry about the disease (49.6%), followed by a substantial impact of diabetes (46.6%) and low life satisfaction (42.9%), as shown in Table 2. The prevalence of depression, anxiety, and stress was observed to be 49.4, 71.7, and 49.8%, respectively (Table 2). Correlation analysis demonstrated a significant relationship between depression, anxiety, and stress and DASS-21 scores with QoL (*p* < 0.001) (Table 3).

**Table 2:** Total score of RV-DQoL and each domains score and incidence of DAS in people with diabetes (n = 251).

Revised DQoL	Mean (SD)	Median	% of subjects > median score
Satisfaction (30 points)	13.14(4.84)	12	46.9%
Impact (20 points)	9.20(3.37)	9	42.9%
Worry (15 points)	6.82(2.78)	6	46.6%
Total (65 points)	29.16(9.23)	27	49.6%
**Variables**	**Categories**	**Frequency**	**Percentage**
**Depression**	Normal	127	50.6
	Mild	32	12.7
	Moderate	38	15.1
	Severe	17	6.8
	Extremely Severe	37	14.7
**Anxiety**	Normal	71	28.3
	Mild	23	9.2
	Moderate	52	20.7
	Severe	29	11.6
	Extremely Severe	76	30.3
**Stress**	Normal	126	50.2
	Mild	21	8.4
	Moderate	40	15.9
	Severe	41	16.3
	Extremely Severe	23	9.2

RV-DQoL = Revised Version of Diabetes Quality of Life, DAS = Depression, Anxiety and Stress.

**Table 3:** Pearson’s correlation coefficient between DASS-21 score and its domain scores and mean score of diabetes quality of life (DQOL) (*n* = 251).

DASS-21	Quality of life	*p* value
	Correlation coefficient (r)	
Depression score	0.637	< 0.001*
Anxiety score	0.643	< 0.001*
Stress score	0.641	< 0.001*
DASS score	0.684	< 0.001*

Initially, univariate analysis was employed to identify predictors of poor QoL. The results indicated a significant correlation between a DASS score > 38 and poor QoL. Participants with a monthly income >10,000 SR, those who were married or divorced, regularly took medication, engaged in regular exercise, were aged over 40, highly educated, and had altered lipid profile and HbA1c showed a reduced likelihood of experiencing poor QoL. Multivariate analysis revealed that a DASS score > 38 and a disease duration of 10–20 years were significantly associated with poor QoL (Table 4).

**Table 4:** Association of different factors with DQOL (Univariate and multivariate logistic regression analysis) (*n* = 251).

Variables	Categories	OR	value of p	95% CI Lower Upper		aOR	*p* value	95% CI Lower Upper	
Gender	Female	*Reference*							
	Male	0.974	0.920	0.576	1.645	1.866	0.111	0.867	4.017
Age groups	Below 30	*Reference*							
	31–40	0.607	0.458	0.162	2.269	0.684	0.686	0.108	4.321
	41–50	0.214	0.003*	0.078	0.585	0.473	0.356	0.097	2.313
	51–60	0.258	0.004*	0.104	0.640	0.453	0.310	0.098	2.093
	61 and above	0.175	0.001*	0.064	0.475	0.292	0.143	0.056	1.515
Marital Status	Single	*Reference*							
	Married	0.238	0.001*	0.100	0.568	0.568	0.439	0.136	2.381
	Widowed	0.389	0.262	0.075	2.027	1.419	0.755	0.157	12.830
	Divorced	0.233	0.041*	0.058	0.945	0.757	0.784	0.104	5.535
Monthly Income	< 5,000 SR	*Reference*							
	5,000–10,000 SR	0.633	0.261	0.286	1.405	0.932	0.889	0.350	2.483
	10,000–15,000 SR	0.358	0.010*	0.163	0.785	0.664	0.469	0.219	2.013
	>15,000 SR	0.180	0.000*	0.085	0.381	0.402	0.096	0.137	1.176
When were you Diagnosed?	< 1 year	*Reference*							
	1–10 years	0.866	0.723	0.392	1.915	2.522	0.107	0.819	7.771
	10–20 years	1.278	0.571	0.548	2.982	3.927	0.027*	1.172	13.160
	> 20 years	1.198	0.735	0.422	3.402	3.126	0.114	0.761	12.842
Higher education	No	*Reference*							
	Yes	0.456	0.007*	0.259	0.805	0.568	0.149	0.264	1.224
Regular Medicine	No	*Reference*							
	Yes	0.362	0.013*	0.163	0.807	0.474	0.168	0.164	1.369
Smoker	No	*Reference*							
	Yes	0.688	0.203	0.387	1.224	0.483	0.068	0.221	1.056
Exercise regularly	No	*Reference*							
	Yes	0.525	0.033*	0.290	0.951	0.971	0.938	0.456	2.067
Lipid profile	Not altered	*Reference*							
	Altered	0.497	0.010*	0.293	0.843	0.755	0.431	0.376	1.519
HbA1c	Not elevated	*Reference*							
	Elevated	0.364	0.000*	0.217	0.609	0.515	0.053	0.263	1.009
DASS Score	≤ 38	*Reference*							
	> 38	5.786	0.000*	3.335	10.039	3.861	0.000*	1.957	7.618

OR = odds ratio, aOR = adjusted odds ratio, *Significant, DASS Score was categorized based on the median score.

In the case of depression, the univariate analysis indicated a significant association with elevated HbA1c levels and DQoL scores >27. Individuals with a monthly income >15,000 SR across all categories of disease duration, regular medication intake, altered lipid profile, and an age above 60 exhibited a reduced likelihood of depression. Multivariate analysis revealed that monthly incomes of 5,000–10,000 SR and > 15,000 SR across all disease duration categories and altered lipid profile were linked to a lower likelihood of depression. Simultaneously, higher education levels and DQoL scores >27 were significantly associated with an increased likelihood of depression (Table 5).

**Table 5:** Association of different factors with depression (Univariate and multivariate logistic regression analysis) (*n* = 251).

**Variables**	**Categories**	OR	*p* value	95% CI Lower Upper		aOR	*p* value	95% CI Lower Upper	
Gender	Female	*Reference*							
	Male	0.964	0.891	0.573	1.624	1.341	0.455	0.621	2.896
Age groups	Below 30	*Reference*							
	31–40	1.981	0.272	0.585	6.705	4.030	0.125	0.679	23.927
	41–50	0.640	0.316	0.268	1.530	2.718	0.228	0.536	13.795
	51–60	0.809	0.583	0.379	1.726	2.475	0.253	0.523	11.717
	61 and above	0.405	0.043*	0.168	0.973	1.665	0.550	0.313	8.853
Marital status	Single	*Reference*							
	Married	0.516	0.072	0.251	1.062	0.690	0.613	0.164	2.901
	Widowed	1.014	0.986	0.209	4.916	1.604	0.686	0.162	15.860
	Divorced	0.852	0.813	0.226	3.209	2.984	0.287	0.398	22.371
Monthly Income	< 5,000 SR	*Reference*							
	5,000–10,000 SR	0.503	0.068	0.240	1.053	0.371	0.039*	0.145	0.953
	10,000–15,000 SR	0.501	0.070	0.238	1.057	0.344	0.056	0.115	1.026
	>15,000 SR	0.344	0.003*	0.171	0.693	0.304	0.029*	0.104	0.884
When were you Diagnosed?	< 1 year	*Reference*							
	1–10 years	0.252	0.002*	0.104	0.609	0.159	0.001*	0.052	0.481
	10–20 years	0.367	0.034*	0.146	0.926	0.218	0.014*	0.065	0.732
	> 20 years	0.323	0.045*	0.107	0.973	0.250	0.050*	0.063	0.999
Higher education	No	*Reference*							
	Yes	1.042	0.881	0.608	1.786	2.243	0.048*	1.006	4.999
Regular Medicine	No	*Reference*							
	Yes	0.322	0.004*	0.148	0.701	0.443	0.105	0.166	1.185
Smoker	No	*Reference*							
	Yes	0.947	0.854	0.534	1.682	1.078	0.847	0.502	2.316
Exercise regularly	No	*Reference*							
	Yes	0.599	0.092	0.329	1.088	0.925	0.844	0.427	2.006
Lipid profile	Not altered	*Reference*							
	Altered	0.483	0.006*	0.287	0.813	0.455	0.022*	0.231	0.895
HbA1c	Not elevated	*Reference*							
	Elevated	0.535	0.014*	0.324	0.882	1.048	0.891	0.539	2.036
DQOL Score	≤ 27	*Reference*							
	> 27	5.283	0.000*	3.066	9.103	5.049	0.000*	2.539	10.040

OR = odds ratio, aOR = adjusted odds ratio, *Significant, DQOL Score was categorized based on the median score.

Regarding anxiety, the univariate analysis demonstrated a significant association between a DQoL score > 27 and anxiety. Individuals with a monthly income of 10,000–15,000 SR and > 15,000 SR, disease duration of 1–20 years, regular medication intake, marriage, age over 40, and altered lipid profile exhibited a reduced likelihood of anxiety. Multivariate analysis indicated that a disease duration of 1–20 years, regular medication intake, and altered lipid profile were associated with a lower likelihood of anxiety. Moreover, a DQoL score > 27 was significantly linked to an increased likelihood of anxiety (Table 6).

**Table 6:** Association of different factors with anxiety (Univariate and multivariate logistic regression analysis) (*n* = 251).

Variables	Categories	OR	value of p	95% CI Lower Upper		aOR	*p* value	95% CI Lower Upper	
Gender	Female	*Reference*							
	Male	0.677	0.203	0.372	1.234	0.591	0.263	0.236	1.484
Age groups	Below 30	*Reference*							
	31–40	0.286	0.194	0.043	1.889	0.893	0.932	0.066	12.013
	41–50	0.118	0.007*	0.025	0.558	0.519	0.561	0.057	4.736
	51–60	0.129	0.007*	0.029	0.570	0.360	0.364	0.040	3.273
	61 and above	0.083	0.001*	0.018	0.384	0.361	0.377	0.038	3.462
Marital status	Single	*Reference*							
	Married	0.051	0.004*	0.007	0.384	0.097	0.098	0.006	1.542
	Widowed	0.194	0.266	0.011	3.490	0.396	0.635	0.009	18.192
	Divorced	0.306	0.415	0.018	5.298	2.175	0.677	0.056	83.836
Monthly Income	< 5,000 SR	*Reference*							
	5,000–10,000 SR	0.415	0.082	0.154	1.119	0.862	0.823	0.236	3.145
	10,000–15,000 SR	0.204	0.001*	0.079	0.530	0.465	0.269	0.120	1.808
	>15,000 SR	0.231	0.002*	0.093	0.577	0.833	0.792	0.215	3.231
When were you Diagnosed?	< 1 year	*Reference*							
	1–10 years	0.212	0.015*	0.061	0.739	0.088	0.004*	0.016	0.469
	10–20 years	0.238	0.029*	0.066	0.862	0.124	0.018*	0.022	0.704
	> 20 years	0.471	0.337	0.101	2.191	0.342	0.282	0.048	2.417
Higher education	No	*Reference*							
	Yes	0.582	0.096	0.308	1.100	0.765	0.577	0.299	1.957
Regular Medicine	No	*Reference*							
	Yes	0.124	0.005*	0.029	0.533	0.087	0.004*	0.017	0.458
Smoker	No	*Reference*							
	Yes	0.953	0.881	0.506	1.795	1.021	0.962	0.423	2.467
Exercise regularly	No	*Reference*							
	Yes	0.557	0.065	0.299	1.037	0.632	0.283	0.273	1.462
Lipid profile	Not altered	*Reference*							
	Altered	0.380	0.003*	0.203	0.714	0.185	0.000*	0.077	0.444
HbA1c	Not elevated	*Reference*							
	Elevated	0.669	0.156	0.385	1.165	1.695	0.198	0.759	3.786
DQOL Score	≤ 27	*Reference*							
	> 27	5.474	0.000*	2.974	10.074	5.196	0.000*	2.306	11.708

OR = odds ratio, aOR = adjusted odds ratio, *Significant, DQOL Score was categorized based on the median score.

In the case of stress, univariate analysis revealed that a DQoL score > 27 was significantly associated with stress. Individuals with a monthly income >5,000 SR, disease duration of 1–10 years, regular medication intake, age over 60, and elevated HbA1c and altered lipid profile displayed a lower likelihood of stress. Multivariate analysis indicated that monthly incomes of 5,000–10,000 SR and > 15,000 SR, disease duration of 1–20 years, regular medication intake, and altered lipid profile were associated with a lower likelihood of stress. Furthermore, being divorced and having a DQoL score > 27 were also significantly associated with a higher likelihood of stress (Table 7).

**Table 7:** Association of different factors with stress (Univariate and multivariate logistic regression analysis) (*n* = 251).

Variables	Categories	OR	value of p	95% CI Lower Upper		aOR	*p* value	95% CI Lower Upper	
Gender	Female	*Reference*							
	Male	0.858	0.564	0.509	1.445	0.970	0.938	0.455	2.069
Age groups	Below 30	*Reference*							
	31–40	2.130	0.252	0.584	7.776	3.089	0.215	0.520	18.350
	41–50	0.609	0.269	0.252	1.468	1.044	0.959	0.206	5.292
	51–60	0.551	0.130	0.255	1.191	0.670	0.610	0.144	3.124
	61 and above	0.323	0.013*	0.133	0.785	0.504	0.417	0.096	2.638
Marital status	Single	*Reference*							
	Married	0.602	0.164	0.295	1.231	2.732	0.158	0.676	11.035
	Widowed	1.136	0.874	0.235	5.487	6.409	0.104	0.684	60.038
	Divorced	0.955	0.945	0.254	3.581	11.235	0.018*	1.526	82.737
Monthly Income	< 5,000 SR	*Reference*							
	5,000–10,000 SR	0.463	0.044*	0.219	0.980	0.375	0.042*	0.146	0.965
	10,000–15,000 SR	0.402	0.018*	0.189	0.855	0.360	0.062	0.123	1.054
	>15,000 SR	0.280	0.000*	0.137	0.571	0.338	0.043*	0.118	0.969
When were you Diagnosed?	< 1 year	*Reference*							
	1–10 years	0.296	0.005*	0.126	0.698	0.165	0.002*	0.052	0.518
	10–20 years	0.456	0.087	0.185	1.121	0.264	0.036*	0.076	0.914
	> 20 years	0.441	0.138	0.149	1.300	0.372	0.172	0.090	1.539
Higher education	No	*Reference*							
	Yes	0.677	0.158	0.393	1.164	1.199	0.651	0.546	2.630
Regular Medicine	No	*Reference*							
	Yes	0.235	0.001*	0.102	0.539	0.256	0.012*	0.088	0.740
Smoker	No	*Reference*							
	Yes	1.200	0.534	0.675	2.131	1.287	0.522	0.594	2.792
Exercise regularly	No	*Reference*							
	Yes	0.586	0.080	0.323	1.065	0.937	0.868	0.435	2.018
Lipid profile	Not altered	*Reference*							
	Altered	0.430	0.002*	0.255	0.727	0.331	0.001*	0.169	0.649
HbA1c	Not elevated	*Reference*							
	Elevated	0.589	0.038*	0.358	0.971	1.182	0.622	0.608	2.300
DQOL Score	≤ 27	*Reference*							
	> 27	4.349	0.000*	2.551	7.414	3.383	0.000*	1.726	6.631

OR = odds ratio, aOR = adjusted odds ratio, *Significant, DQOL Score was categorized based on the median score.

## Discussion

The results of the present study showed that 46.9% of individuals with DM had poor QoL. Almost half of the individuals (49.6%) reported high worry about the disease, followed by a high diabetes impact (46.6%) and low life satisfaction (42.9%). The present study results regarding DQoL and its domains are similar to a recent Nigerian study that reported 43% had poor QoL, 56.3% had a high diabetes impact, 53% had low life satisfaction, and 32.7% had high worry about diabetes (20). There are variable reports about the QoL among individuals with DM from low to moderate (21), poor (22, 23), good (24), and moderate HRQoL.

Literature indicates that the QoL among diabetic people can be related to various causes, including using different QoL questionnaires, differences in participants’ educational and socioeconomic status, disparities in age, societal norms, availability of healthcare facilities, and comorbidities. These factors can impact diabetic patients’ subjective experiences and general well-being, making it challenging to compare QoL outcomes across research. It underlines the necessity of considering these characteristics when evaluating and comparing QoL outcomes and urges individualized healthcare interventions to accommodate the varied requirements of diverse communities.

Our study results indicated that many factors could contribute to a lower risk of poor QoL in DM individuals, including the participants’ higher monthly income, being married and divorced, taking the medication regularly, exercising regularly, being highly educated, being older, and having elevated HbA1c and altered lipid profile. Moreover, increased depression, anxiety, and stress scale score (> 38) and longer disease duration were significantly associated with poor QoL. The present findings are more or less similar to other studies (15, 20). Al-Qerem W (2021) showed an association of the mean DQoL questionnaire total score with HbA1c, insulin treatment, diabetic complications, and the low-income group (15). Education, marital status, profession, parental history of diabetes, comorbidities, and social support were all linked to DQoL in a Nigerian study (20).

Our results regarding the association of HbA1c and QoL contrast with a few other studies (25–27). There is a strong likelihood that better QoL contributes to improved self-care behaviors, leading to lower HbA1c levels (25).

Moreover, DM restrictions impact QoL. A recent study among individuals from Saudi Arabia and Pakistan reported depression among DM subjects due to restrictions, and they admitted that they could not enjoy the Eid al Fitr festivity and felt depressed (26). A systematic review indicated that depression and anxiety in patients reduce their perception of QoL (27). QoL has a strong influence on the lives of individuals with diabetes and has a direct relationship with glycemic control (28, 29). Therefore, we cannot manage an individual with DM without improving QoL. Our findings revealed a significant positive relationship between QoL and depression, anxiety, and stress scores. It appears that depression, anxiety, and stress are dependent on QoL and vice versa.

Similar to our results, a study reported that DM subjects who were educated, married, had a family history of diabetes, and had social support had a lower probability of poor QoL (20). We do not have an appropriate explanation for elevated HbA1c and altered lipid profile association with a lower probability of poor QoL. The current study did not measure participants’ lipid profiles and HbA1c, which were self-reported; there could have been any mistake in the grasp of this question.

A study reported that individuals aged ≤59 years, with higher annual income, living alone or with a spouse, people with regular exercise, a high level of knowledge regarding prevention and care, no diabetic complications, and those whose medical fees were supported by the government had a greater probability of having good QoL (21).

The present study results observed that DM subjects aged 41–50, 51–60, and > 60 years had a lower chance of poor QoL compared to the <40 years age group. Our findings contrast with other studies that reported QoL decreased significantly with age in DM patients (21, 30, 31). Few studies also reported a higher QoL score among younger people (32, 33). An Iranian systematic review reported poor QoL results associated mainly with Iranian patients (women) with worse disease control (higher HbA1c values) and advanced age with higher complications and lower socioeconomic level (34).

Our study results indicated that participants whose monthly income was >10,000 SR had a lower chance of poor QoL. A Saudi study reported that a monthly income of less than 5,000 SR is associated with lower HRQoL (1). Our results observed that highly educated individuals had a lower chance of poor QoL. Our results are consistent with Omani, and Saudi studies that reported diabetic people with higher education had better HRQoL (1, 35).

The findings mentioned above could be explained by the fact that individuals with high incomes may have better diabetes control due to their educational attainment and access to quality medical care resources. In this instance, they can take actions such as regularly following their doctor’s prescription while embracing healthy lifestyle choices without being concerned about the costs involved with critical medical prescriptions. However, health outcomes continue to be multi-dimensional due to various factors such as a family history of chronic illnesses, access to or lack of access to essential healthcare services, and individual behaviors, among others, all of which affect overall wellness.

Our study found no association of gender with QoL, while few studies reported poor QoL in females (1, 33, 36). Such disparities may be due, in part, to wider gender disparities in some societies (36), which may have been less visible in Saudi Arabia. Our findings can be explained by the point that in KSA, the literacy rate among both genders is approximately 98–99%. Both have equal access to healthcare facilities and information hubs. They know controlling their blood sugar levels depends on proper treatment and a healthy lifestyle. The current study differs from others in the association between socioeconomic status and other variables with QoL due to using different questionnaires to measure QoL among people with DM, population socioeconomic status, educational level, and healthcare facility availability.

The incidence of depression, anxiety, and stress was 49.4, 71.7, and 49.8%, respectively. There was a strong relationship between depression, anxiety, and stress and DASS-21 scores with QoL. This showed the presence of higher DAS levels among Saudi people with diabetes, and these also affect their QoL. The prevalence of depression, anxiety, and stress among our study participants was higher than in several other studies (37, 38). Another Saudi study reported 33.8, 38.3, and 25.5% depression, anxiety, and stress prevalence, respectively (39). A recent Ecuadorian survey reported the prevalence of depression, anxiety, and stress among diabetic individuals at 31.7, 33.7, and 25%, respectively (40).

Our study reported a 50% frequency of depression among people with diabetes, while Indian research reported a 41% prevalence of depression among people with diabetes. The study recommended that the assessment of depression be done in every diabetic subject, irrespective of other factors, since the rate of depression among people with diabetes remains high (41). A recent British study also reported prevalence of anxiety and depression among diabetes individuals.

In the present investigation, there was no relationship between gender and depression, anxiety, and stress. In contrast to our findings, a few studies have found the female gender to be a predictor of DAS (38–40). Depression, anxiety, and stress among females has been linked to a lack of social support and unpleasant life events (42, 43).

People living with diabetes exhibit variations in depression and anxiety. Discrepancies in lifestyle habits, healthcare management systems/resources, availability of treatment, and management in underdeveloped, developing, and developed countries may explain such discrepancies. Screening equipment and sample methods may also reflect diabetes depression and anxiety demographics.

Our study findings showed that a poor QoL score (DQOL scores >27) was significantly associated with depression, anxiety, and stress. The present study also found that participants with a higher monthly income, shorter disease duration, regular medicine use, altered lipid profiles, and older age had a lower chance of depression, anxiety, and stress. Individuals with elevated HbA1c had a lower risk of depression and stress.

Our results indicated that higher income and adherence to medication were associated with a lower chance of depression, anxiety, and stress. A few studies have reported that compliance with treatment (39) and higher income (37, 44) were associated with decreased odds of depression, anxiety, and stress. Financial constraints can be linked to increased stress levels in coping with poverty, leading to an increase in the risk of anxiety.

In our study, all duration categories of the disease (since T2DM diagnosis) had a lower probability of depression. These results contrast with a few other studies (35, 45, 46). This might be attributed to the Kingdom’s 98–99% literacy rate, along with favorable socioeconomic conditions. All resources are within their reach. Moreover, nowadays, all knowledge resources are at the fingertips of individuals because of smartphones. In the KSA, healthcare is the responsibility of the Kingdom, and all healthcare facilities, including medicine, are free for all Saudi nationals. Contradictory to our finding, an increased duration of diabetes was also associated with increased anxiety and depressive symptoms because, as the duration of diabetes increased, the financial burden of healthcare expenditure and management of diabetes-related complications in the long term led to psychological illnesses (47).

Our study observed that being older was a protective factor against depression, anxiety, and stress. Others reported similar findings (39, 48, 49). The most likely reason could be that older people usually have a longer duration of diabetes, so with time, they accept all the related issues. They compromise with DM and have learned to live with it. Therefore, the depression, anxiety, and stress level is lower among older people than younger individuals.

The present study found that individuals with DM who had an altered lipid profile had a lower probability of depression, anxiety, and stress. Our finding contradicts several other reports (37, 39, 45, 46). The current study’s inconsistent findings could be attributed to the study’s reliance on self-reported data, the likelihood of participants misinterpreting or selecting incorrect options, and other confounding factors. More analysis using direct measurements and a more detailed methodology is required to elucidate the association between an altered lipid profile, higher HbA1c, and depression, anxiety, and stress scores in individuals with diabetes.

Due to various sociodemographic and clinical factors, a multidisciplinary team-based strategy is necessary to successfully screen and treat psychosocial disorders in T2DM patients. In conclusion, depression, anxiety, and stress were common in T2DM patients. Thus, all these factors should be considered when monitoring T2DM patients.

There is a need to reassure individuals with diabetes that nutrition, exercise, medication, and stress management may lead to a long and healthy life. Individuals with diabetes should attend sessions regarding depression, anxiety, and stress management, improving QoL, and lifestyle modifications. Depression, anxiety, and stress affect their blood sugar control and impairs their QoL. They should sleep 6–8 h daily, exercise 30–60 min regularly, eat a balanced diet, check their blood sugar, and undergo lab tests regularly. A psychiatrist can help individuals with depression, anxiety, and stress in preventing short– and long-term consequences.

The study’s findings suggest poor QoL and a higher prevalence of DAS among T2DM individuals. Several factors can contribute to a lower risk of depression, anxiety, and stress in people with DM, including a higher monthly income, regular medication use, altered lipid profile, and older age. Our findings will help develop efficient intervention programs to enhance T2DM-related QoL in individuals with diabetes. Such initiatives should focus on groups with low education, lower-income individuals, younger age groups, unmarried individuals, those with low physical activity levels, irregular exercise, and individuals with 10–20 years of diabetic duration.

## Limitations and recommendations of the study

This study is not without limitations. Concerning the study design, we did not compare the findings reported among people with diabetes with non-diabetic controls, nor did we introduce any intervention and compare the parameters before and after the intervention. The limited sample size and the conduction of the study at a single center were also some of the study’s limitations. Our participants’ mean age was 50, which means our participants were not very old, and QoL and depression, anxiety, and stress can differ in older people. This study did not account for personality traits. It could be a limitation because personality features can contribute to anxiety and depression. Therefore, a multi-centered comparative study with a larger sample size and participants from multiple nationalities would yield more comprehensive findings than observed in this study.

The present study findings indicate that individuals with diabetes face significant challenges that impact their overall QoL. We observed a higher prevalence of depression, anxiety, and stress in the study population, indicating a strong relationship between diabetes and psychological well-being. These findings underscore the importance of a multifaceted approach to diabetes management that includes physical and mental health support. Enhancing diabetic individuals’ QoL involves comprehensive care that addresses both the medical aspects of the condition and the patient’s emotional well-being. Recognizing and addressing the psychological impact of diabetes allows healthcare practitioners to help patients better control their disease and improve their overall QoL.

## Conclusion

The current study’s results revealed that approximately half of the individuals with T2DM had poor QoL, and the prevalence of depression, anxiety, and stress were 49.4, 71.7, and 49.8%, respectively. The mean domain scores for impact, worry, and satisfaction of RV-DQoL were not up to the mark. Several factors were associated with QoL and depression, anxiety, and stress; furthermore, there was an association between DASS-21 and QoL scores. Longitudinal studies are needed to explore the relationship between QoL and depression, anxiety, and stress and their impact on individuals with diabetes.

## Data availability statement

The original contributions presented in the study are included in the article/Supplementary material, further inquiries can be directed to the corresponding author.

## Ethics statement

The studies involving humans were approved by Ethical approval was obtained from the Biomedical Ethics Research Committee of King Abdulaziz University, Jeddah (Reference No. 449-19). 'Written informed consent was obtained from the individuals for the publication of any potentially identifiable data included in this article'. The studies were conducted in accordance with the local legislation and institutional requirements. The participants provided their written informed consent to participate in this study.

## Author contributions

SA: Conceptualization, Investigation, Resources, Writing – original draft. MB: Formal analysis, Investigation, Methodology, Writing – review & editing. WA: Writing – review & editing, Project administration, Resources, Supervision. ZG: Project administration, Writing – review & editing, Investigation, Methodology. MA-H: Methodology, Writing – review & editing, Formal analysis, Resources. AB: Formal analysis, Methodology, Resources, Writing – review & editing.
